# An ambient stable core-substituted perylene bisimide dianion: isolation and single crystal structure analysis†
†Electronic supplementary information (ESI) available: Synthesis, spectroelectrochemistry, UV-vis, electrochemistry, X-ray diffraction, NMR and mass spectra. CCDC 1032959. For ESI and crystallographic data in CIF or other electronic format see DOI: 10.1039/c4sc03671a



**DOI:** 10.1039/c4sc03671a

**Published:** 2015-01-16

**Authors:** Sabine Seifert, David Schmidt, Frank Würthner

**Affiliations:** a Universität Würzburg , Institut für Organische Chemie and Center for Nanosystems Chemistry , Am Hubland , 97074 Würzburg , Germany . Email: wuerthner@chemie.uni-wuerzburg.de

## Abstract

Here we report the first example of an isolable, ambient stable perylene bisimide (PBI) dianion which was synthesized by catalytic reduction of a highly electron deficient PBI derivative. The remarkable stability of this unprecedented dianion in air for months facilitated its complete characterization by different methods, including single crystal X-ray analysis. Furthermore, solvent dependent cyclic and square wave voltammetry studies revealed that the formation of PBI dianions is preferred in more polar solvents, whereas the generation of PBI radical anions should be favoured in less polar solvents.

## Abstract 2

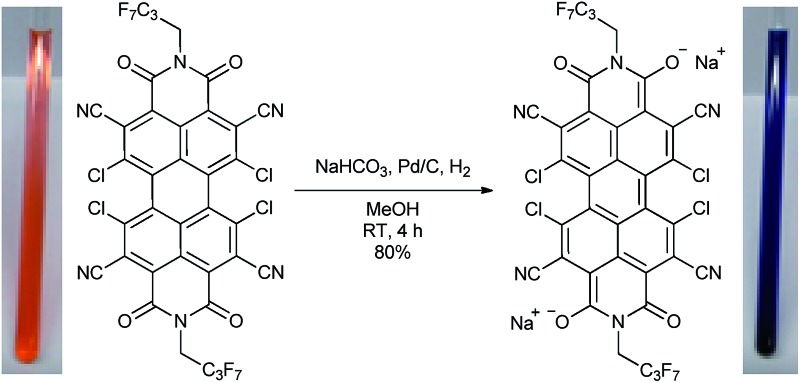
The reduction of a highly electron deficient PBI afforded the corresponding dianion disodium salt that was characterized by single crystal structure analysis.

## Introduction

Perylene bisimide (PBI) dyes are one of the most widely studied organic colorants and may be considered as the archetype for an electron poor π-conjugated aromatic scaffold.^1^ More recently, the interest in this class of compounds as an organic alternative for the common inorganic semiconducting materials has grown tremendously.^2^ In particular, the molecular design that is composed of two highly electron withdrawing imide subunits attached to a polycyclic aromatic core that can be functionalized easily appears to be very promising to shift the energy level of the lowest unoccupied molecular orbital (LUMO) towards high performance and even air-stable n-type organic semiconductors. Furthermore, the absorption and emission wavelengths of these systems can be adjusted within the visible or even near infrared region which is a prerequisite for many optoelectronic applications.^2^ Interestingly, the first application of these unique molecules as vat dyes^3^ – similar to indigo^4^ – has already been based on the possibility to reduce the water insoluble PBI derivatives to the corresponding water soluble anions (leuko bases). Related to this initial coloration process the most recent implementation of PBIs in organic field effect transistors (OFETs)^5^ and organic photovoltaics (OPV)^2,6^ benefits in a similar way from the appreciable stability of reduced PBI species^7^ where charge carriers are either injected from the source electrode or generated by photoinduced electron transfer processes at a dielectric interface. Moreover, there is some recent effort to use multiple reduced rylene bisimide derivatives as rechargeable supramolecular materials or as organic alternatives for common lithium-ion batteries.^8^ Regarding the importance of reduced PBI species in all these applications, it is rather surprising that only very recently the first stable and completely characterized PBI radical anion could be achieved,^9^ and to date not a single example of an isolable, ambient stable perylene bisimide dianion is known. The stability of PBI dianions in reducing environments has been demonstrated by the utilization of these dyes as vat dyes^3,10^ and recent compelling studies with regard to their physical properties and electronic structure. Thus, Rybtchinski and co-workers reported that a core-unsubstituted PBI containing polyethylene glycol chains at the imide positions could be reduced with sodium dithionite (Na_2_S_2_O_4_) in water to its dianion, which was stable for months in deoxygenated aqueous solution due to the delocalized aromatic character.^7c,d^ Similarly, Brochsztain *et al.* have investigated the aggregation behaviour of a PBI dianion that was generated by Na_2_S_2_O_4_ titration in water–ethanol mixtures.^7a,b^ Although PBI anions and/or dianions are discussed to be sufficiently stable in solution and within the (opto)electronic device by virtue of a robust and protecting environment, the isolation of such an ambient stable compound appears to be rather difficult as reduced PBI species are prone to react with moderate oxidants. In this regard we thought that recently reported highly electron deficient PBIs such as octachloro-,^11^ tetracyano-^12^ and in particular tetrachloro-tetracyano-PBI,^13^ bearing multiple electron withdrawing substituents should be suitable to stabilize the respective PBI anions and/or dianions. Indeed, for the tetrachlorotetracyano-PBI Wang and co-workers recently reported two reversible reduction processes with potentials of *E*_1/2_ (PBI/PBI˙^–^) = –0.20 V and *E*_1/2_ (PBI˙^–^/PBI^2–^) = –0.54 V (*vs.* ferrocene/ferrocenium) that are shifted about 800–900 mV to more positive values than those for the parent PBI.^13^ Therefore, such highly electron deficient PBIs are promising precursors for ambient stable PBI dianions.

Pleasingly, our presumption has been shown to be viable, and we report here for the first time the synthesis and isolation of an ambient stable PBI dianion disodium salt from a tetrachlorotetracyano-PBI derivative. Moreover, we have investigated the optical and electrochemical properties as well as the solid-state structural features of this unique PBI dianion.

## Results and discussion

### Synthesis

**Scheme 1:**
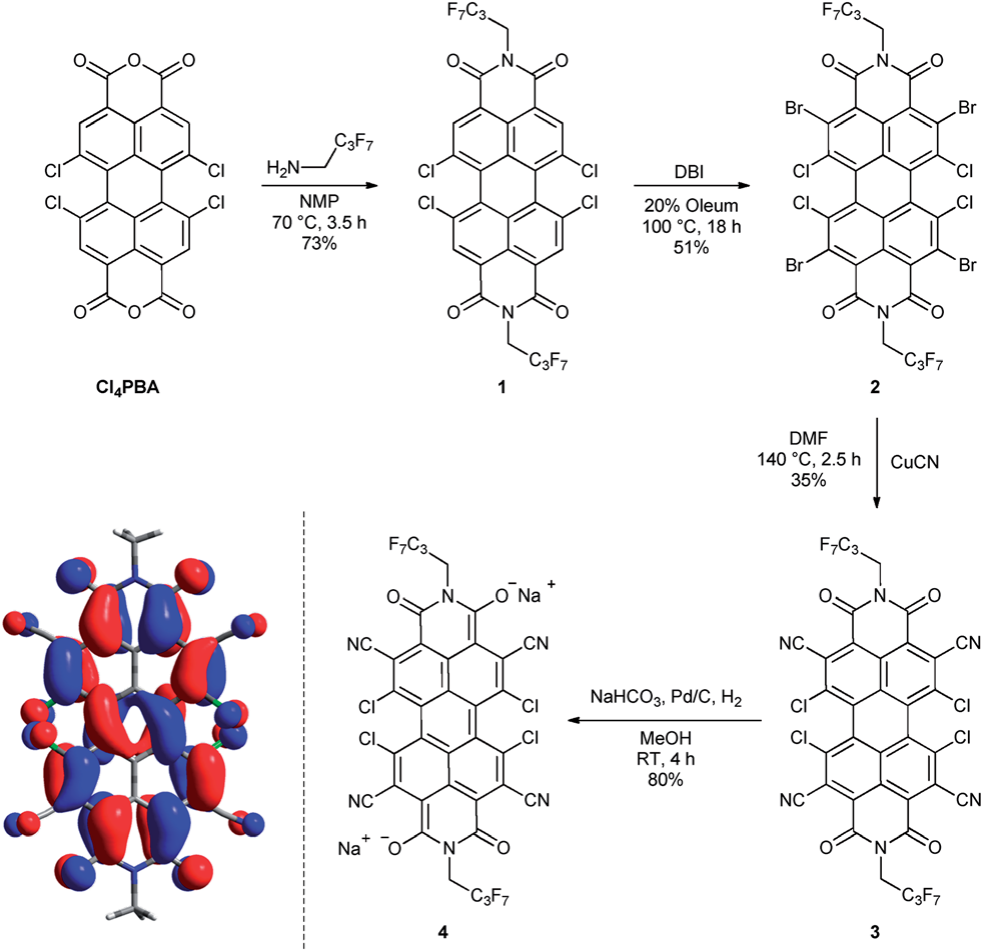
Synthesis of the core-substituted PBIs **1–3** and the PBI dianion disodium salt **4**. Left: visualization of the calculated HOMO (B3LYP, 6-31+G*, isoval 0.02 a.u.) of a simplified model system of **4** where the imide substituents were replaced by methyl groups and the sodium cations were ommited. Whilst for simplicity a single resonance structure is depicted for **4** the calculation reveals the delocalization of the negative charge over the entire π-conjugated scaffold.

Starting from 1,6,7,12-tetrachloroperylene-3,4:9,10-tetracarb-oxylic acid bisanhydride (Cl_4_PBA)^14^ the PBI dianion disodium salt **4** was synthesized in four steps (Scheme 1). After imidization of Cl_4_PBA with heptafluorobutylamine,^5*b*^ Cl_4_PBI **1** was brominated using dibromoisocyanuric acid (DBI) in oleum (20%) to yield Br_4_Cl_4_PBI **2** in 51%. Subsequent nucleophilic substitution of all four bromine atoms in Br_4_Cl_4_PBI **2** with cyano groups using CuCN as the cyanide source, following a slightly modified literature procedure,^13^ afforded the highly electron deficient (CN)_4_Cl_4_PBI **3**. Finally, tetrachlorotetracyano PBI **3** was reduced by hydrogen in the presence of 10% (wt) palladium on activated charcoal (Pd/C) and an excess of sodium hydrogen carbonate to the desired PBI dianion disodium salt **4**. Immediately after replacing the nitrogen atmosphere by hydrogen an intensive color change of the reaction mixture from orange to dark blue was observed. After filtration of the residual catalyst and precipitation of the product out of an acetone solution by adding pentane, **4** could be isolated as analytical pure material in 80% yield.

Although this mild catalytic procedure has initially been introduced for the reductive dehalogenation of aryl halides,^15^ a simultaneous dechlorination during the reduction of PBI **3** to **4** was not observed under the applied reaction conditions. This finding is in contrast to our observation made in analogous reduction experiments with PBI **1** and **2** under the same reaction conditions. Although a similar color change from orange to blue was observed in both cases, PBI **1** was reisolated after work-up in ambient atmosphere, whereas for PBI **2** a debromination to Cl_4_PBI **1** was observed. These significant differences regarding the reactivity of PBIs **1**, **2** and **3** towards catalytic reduction elucidate that the appropriate choice of electron withdrawing substituents in PBIs is of great importance for the stabilization of perylene bisimide dianions.

### Structure elucidation

**Fig. 1:**
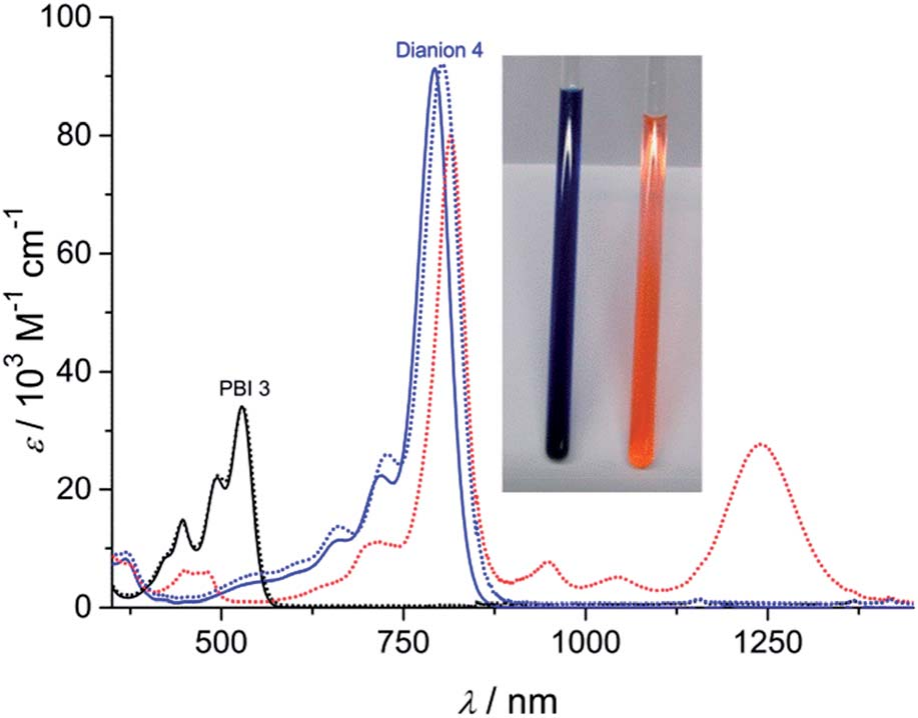
UV-vis absorption spectra (25 °C, *c* = 2 × 10^–5^ M, ambient conditions) of the neutral PBI **3** in dichloromethane (black solid line), the dianion disodium salt **4** in acetone (blue solid line) and the corresponding electrochemically generated radical anion **3**˙^–^ (dotted in red) and dianion **3** ^2–^ (dotted in blue). Inset: photograph of the solutions of PBI **3** in dichloromethane (right) and PBI dianion disodium salt **4** in acetone (left).

The dianion disodium salt **4** was unequivocally characterized by multinuclear NMR spectroscopy, high resolution mass spectrometry, elemental analysis as well as single crystal X-ray diffraction experiments. The UV-vis absorption spectrum of compound **4** features a very intensive absorption band within the NIR region at around 793 nm that is significantly shifted to longer wavelength compared with the neutral starting material **3** (Fig. 1). This very characteristic absorption band is in excellent agreement with electrochemically generated PBI **3** ^2–^ (Fig. 1 and S1†), but considerably shifted to higher wavelength in comparison to core-unsubstituted PBI dianions.^7b,c^ Since an increasing number of electron withdrawing groups at the aromatic core of PBI dianions causes a continuous bathochromic shift of the absorption maxima,^7e,9^ the red-shift of the absorption bands of compound **4** can be attributed to the four chlorine and four cyano substituents. Whereas the absorption band of **4** exhibits a vibronic fine structure that is well resolved like the one of parent PBI **3**, the absorption coefficient of the dianion disodium salt **4** is considerably increased from 34 100 M^–1^ cm^–1^ (PBI **3**) to 91 300 M^–1^ cm^–1^.

Unchanged spectral features can be observed for isolated **4** after exposing the solid material for approximately 5 months to air and moisture (ambient conditions) upon dissolution in acetone. The acetone solution itself also remains stable for weeks under ambient conditions since no spectral changes can be observed. These findings corroborate the remarkable stability of the PBI dianion disodium salt in solution as well as in the solid state under ambient conditions.

### Crystallographic analysis

**Fig. 2:**
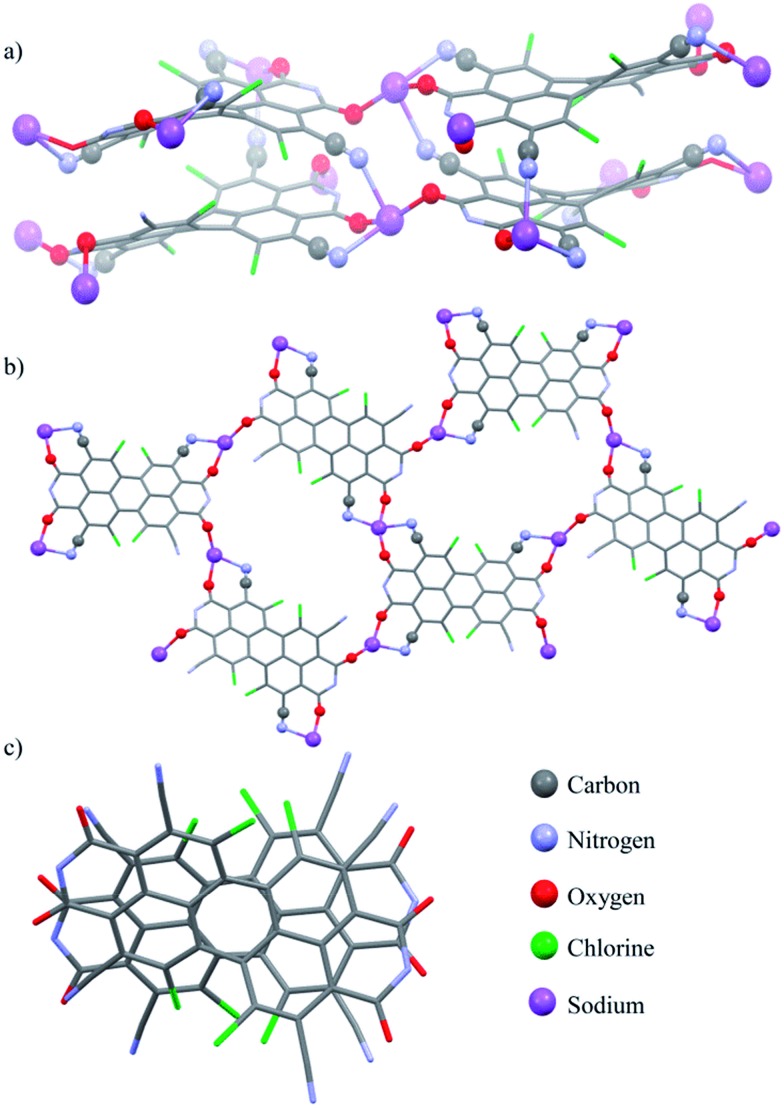
(a) Molecular structure of the PBI dianion disodium salt **4** in the solid state, (b) top view on the perylene cores illustrating the connectivity, (c) visualization of the rotational displacement (fluorinated alkyl chains and solvent molecules are omitted for clarity).

Single crystals of perylene bisimide dianion disodium salt **4** were grown by slow diffusion of pentane into its 1 mM acetone solution to unambiguously determine the structure and the connectivities of this novel compound within the solid state. This dianion disodium salt crystallizes in the triclinic space group *P*1 including both atropo-enantiomers (*P* and *M*) within the asymmetric unit (Fig. 2, S6 and S7†). The packing motif of **4** is characterized by bridging sodium cations that are coordinated by the carbonyl and cyano groups of up to three PBI molecules and a varying amount of solvent molecules (acetone) which help to saturate the coordination sphere of the sodium cations. Since every PBI molecule is equipped with four carbonyl oxygen atoms that are all orientated in different directions, the coordination of sodium by the carbonyl groups of **4** results in a two-dimensional sheet-like structure (Fig. 2b). Each two of these sheets are further interconnected by the additional coordination of cyano groups from the adjacent layer to the sodium cations to form a dimeric layer-type structure (Fig. 2a) with a π–π-distance of 4.33 Å between the PBI centroids. Within these bilayers the PBI molecules are arranged parallel with a rotational displacement of 22° between each two PBI dianions (Fig. 2c). Such an arrangement of PBIs in the solid state is rather scarce since the common structural feature of bay-substituted perylene bisimides with twisted aromatic cores is characterized by transversal displacements along the short and the long axes.^5b,16^ For the given PBI salt an extended three dimensional interconnection is prevented by the incorporation of additional pentane molecules between every bilayer (Fig. S7†). Comparing all bond lengths and angles of the PBI dianion disodium salt **4** with a neutral dibutyl substituted tetrachlorotetracyano PBI derivative^13^ only minor deviations can be ascertained (Fig. S6 and Table S2†).

All these very small changes that affect in particular the carbonyl bonds and the peripheral carbon–carbon bonds of the aromatic core are, however, in very good agreement with theoretical considerations made by Rybtchinski and co-workers, for the parent core-unsubstituted PBI dianion.^7c,7d^ According to their theoretical investigations on a simplified model system the peripheral C=C bonds of perylene bisimide dianions should possess alternating bond distances as it is indeed observed for **4** in the solid state. Whereas the C=C bonds that are arranged longitudinal to the long axis of the PBI core are slightly shortened compared with the neutral dibutyl substituted tetrachlorotetracyano analogue, the C=C bonds that are aligned transversal to this axis are somewhat elongated. Furthermore, upon reduction of PBI **3** to the corresponding dianion disodium salt **4** the twist of the aromatic core is reduced about 2.3° from 35.0 and 36.4° for the neutral dibutyl tetrachlorotetracyano PBI derivative^13^ to 32.9° and 33.9° in **4**. The contraction of the peripheral C=C bonds (longitudinal ones) in PBI dianions has been attributed to the additional amount of electron density that is partially localized on these bonds and is consistent with the calculated HOMO (highest occupied molecular orbital, Scheme 1) of a simplified model system of **4** (heptafluorobutyl chains were replaced by methyl groups, sodium cations were omitted) in which the maximum orbital coefficients can be found on the relevant bonds.^7c,d^ Furthermore, some of the additional electron density of **4** is localized in the C=O and C≡N π* orbitals according to IR spectroscopy (Fig. S8 and S9†). Upon reduction, both, the C=O and C≡N stretch vibrations of **3** are somewhat shifted from 2227 (*ν̃*_C≡N_), 1733 and 1724 (*ν̃*_C=O_) cm^–1^ to 2222 (*ν̃*_C≡N_), 1631 and 1620 (*ν̃*_C=O_) cm^–1^ in **4**. This characteristic shift to lower wavenumbers is accompanied by a significantly increased intensity for the C≡N stretch vibration that is in very good agreement with our calculations (≈factor 400).

### Electrochemical studies

Cyclic voltammetry has been performed on Br_4_Cl_4_PBI **2** and (CN)_4_Cl_4_PBI **3** (and compared to Cl_4_PBI **1**)^5*b*^ in dichloromethane to quantify the influence of the four cyano substituents in PBI **3** on the electron accepting properties within a series of heptafluorobutyl substituted perylene bisimides (Fig. S5 and Table S1†). Two reversible reduction waves with potentials of *E*_1/2_ (PBI/PBI˙^–^) = –0.74 V and *E*_1/2_ (PBI˙^–^/PBI^2–^) = –0.95 V (*vs.* ferrocene/ferrocenium) are observed for Cl_4_PBI **1** ^5*b*^ that become significantly shifted to more positive potentials for the fourfold brominated Br_4_Cl_4_PBI **2** (*E*_1/2_ (PBI/PBI˙^–^) = –0.49 V and *E*_1/2_ (PBI˙^–^/PBI^2–^) = –0.67 V). In the case of the tetrachloro-tetracyano PBI **3** a further shift of both reversible reduction waves to *E*_1/2_ (PBI/PBI˙^–^) = –0.07 V and *E*_1/2_ (PBI˙^–^/PBI^2–^) = –0.41 V was observed confirming the highly electron deficient character of **3**. Both reduction potentials of **3** are even more positive than the ones reported by Wang and co-workers (*vide supra*) and elucidate the electron withdrawing effect of the highly fluorinated butyl substituent on the redox behavior of PBIs.^5b,13^ In fact, to the best of our knowledge, PBI **3** can be regarded as the most electron poor rylene bisimide derivative that has been reported to date.^13,17^

According to these electrochemical investigations the reduction of (CN)_4_Cl_4_PBI **3** to the corresponding PBI radical anion **3**˙^–^ and dianion **3** ^2–^ in dichloromethane can also be monitored by spectroelectrochemistry (Fig. S1†). Upon applying a negative potential the characteristic absorption bands at around 528 nm for the neutral PBI **3** slowly decrease whereas new equally characteristic absorption bands within the NIR region at around 1241, 1046, 948, 815 and 715 nm increase which can be attributed to the formation of the radical anion **3**˙^–^. However, upon performing the second electrochemical reduction to generate the corresponding dianion **3** ^2–^ absorption bands at 804, 728 and 661 nm increase at the expense of those of **3**˙^–^, which are perfectly consistent with the ones of the isolated PBI dianion disodium salt **4** (Fig. 1). Both distinct reduction processes are characterized by several isosbestic points (Fig. S1†) indicating the excellent stability of both, the radical anion and the dianion in less polar solvents like dichloromethane.

**Fig. 3:**
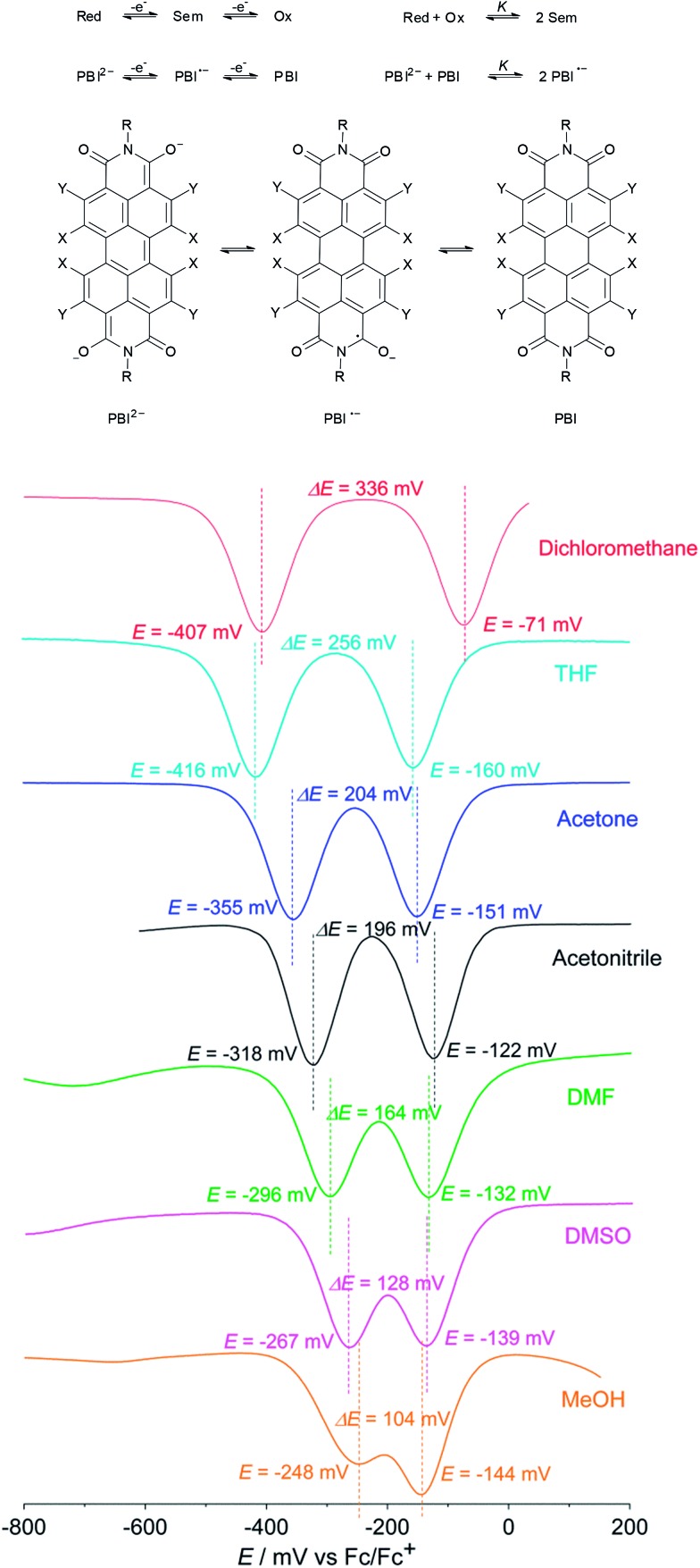
Top: schematic representation of the equilibrium between reduced form (Red ≙ PBI^2–^), intermediate (Sem ≙ PBI˙^–^) and fully oxidized form (Ox ≙ PBI). Bottom: square wave voltammograms of PBI **3** in solvents of different polarity. The dianion disodium salt **4** was used in the case of MeOH for solubility reasons. Reference electrode: Ag/AgCl, working and auxiliary electrode: Pt; 0.1 M TBAHFP, Fc/Fc^+^, 25 °C, *c* ∼ 2 × 10^–4^ M.

Even though the data obtained by cyclic voltammetry and spectroelectrochemistry suggest that the corresponding radical anion should, in principle, also be isolable at least from non-polar solvents, we could just recover the neutral PBI **3** under the same reductive reaction conditions using dichloromethane as a solvent. Apparently, PBI **3**˙^–^ is not stable under ambient conditions. To gain some insights into the stability of PBI radical anions we became interested in a quantitative analysis of the thermodynamic stability of PBI **3**˙^–^. Based on the work of Hünig and co-workers^18^ and a theory of Michaelis and Schubert^19,20^ that is concerned with the determination of semiquinone formation constants *K* to quantify the stability of semiquinones with regard to their disproportionation into quinones and hydroquinone anions in solution, we performed solvent dependent cyclic voltammetry and square wave voltammetry to estimate *K* for PBI **3**˙^–^ (Fig. 3). Using dichloromethane a semiquinone formation constant *K* of 400 000 was calculated that is consistent with two well separated reduction processes. However, the formation of the radical anion PBI **3**˙^–^ becomes less favoured by increasing the polarity of the solvent as demonstrated by a continuously decreased semiquinone formation constant (*K* = 18 500 (THF) > 2500 (acetone) > 1800 (acetonitrile) > 500 (DMF) > 100 (DMSO)). These findings are in very good agreement with the direct formation of the PBI dianion disodium salt **4** in methanol (used as solvent in synthesis) where both reduction processes with a semiquinone formation constant of *K* = 50 (determined using **4** due to solubility reasons) are obviously superimposed (Fig. 3). A distinct protic effect on this behaviour seems to be unlikely as identical reduction potentials were observed by pH dependent measurements in THF (Fig. S4†). In agreement with previous results by Rybtchinski the stabilization of the PBI dianion by protic solvents *via* hydrogen bonding can be confirmed.^7*c*^

## Conclusions

In summary, we reported a straightforward synthetic procedure for the preparation of the first ambient stable perylene bisimide dianion disodium salt using a highly electron deficient perylene bisimide derivative for the stabilization of the additional negative charges. This unprecedented molecule has been fully characterized by UV-vis absorption and NMR spectroscopy as well as single crystal X-ray diffraction experiments. Semiquinone formation constants that have been investigated by cyclic and square wave voltammetry for the PBI radical anion **3**˙^–^ in different solvents reveal that the formation of dianionic perylene bisimides is generally preferred in more polar solvents, whereas the generation of PBI radical anions should be favoured in less polar solvents. The enhanced thermodynamic stability of the PBI dianion in polar media sheds light on the formation and persistence of leuko bases in aqueous media which might also apply to other vat dyes such as BASF's famous indanthrene dyes and indigo.^4,21^
