# Supplementary material for: An ambient stable core-substituted perylene bisimide dianion: isolation and single crystal structure analysis
Source: Chem Sci. 2015 Jan 16;6(3):1663–7. doi: 10.1039/c4sc03671a (PMC5501005; doi:10.1039/c4sc03671a)
Supplement: Supplementary file 1 [file SC-006-C4SC03671A-s001.pdf]

Electronic Supplementary Material (ESI) for Chemical Science.
This journal is © The Royal Society of Chemistry 2015

Electronic Supplementary Information

An ambient stable core-substituted perylene bisimide dianion

*Sabine Seifert, David Schmidt and Frank Würthner**

Universität Würzburg, Institut für Organische Chemie and Center for Nanosystems Chemistry,
Am Hubland, 97074 Würzburg, Germany
E-mail: wuerthner@chemie.uni-wuerzburg.de

Table of Contents

1. Materials and Methods 2
2. Synthesis 3
3. Spectroelectrochemical measurements and UV-Vis-NIR absorption spectra 6
4. CV and SW data 7
5. Single crystal data 10
6. IR spectra 13
7. ^{1}H and ^{13}C NMR 15
9. HRMS spectra 23
10. References 24

1. Materials and Methods

Starting material Cl_4PBA was purchased from SYNTHON Chemicals and its purity was carefully checked by NMR and HRMS. DBI was synthesized according to literature known procedure.[S1] Column chromatography was performed on silica gel (particle size 0.040−0.063 mm). All other commercially available reagents and solvents were of reagent grade and used without further purification. The solvents for the spectroscopic measurements and other experiments were of spectroscopic grade and used without further purification. UV-Vis absorption spectra were recorded on a Perkin Elmer Lamda 950 spectrometer. Steady-state fluorescence spectra were recorded under ambient conditions on a PTI QM4-2003 fluorescence spectrometer and corrected against photomultiplier and lamp intensity. The fluorescence quantum yields were determined by optical dilute method[S2] ($A < 0.05$) using N,N´-di(2,6-diisopropylphenyl)-perylene-3,4:9,10-tetracarboxylic acid bisimide (φ_{fl} = 1.00 in chloroform) as reference. NMR spectra were recorded on a Bruker DMX 400 spectrometer. ^{13}C NMR spectra were broad-band proton-decoupled ($^{13}C\{^1H\}$). Chemical shifts (δ) are listed in parts per million (ppm) and are reported relative to tetramethylsilane. Coupling constants (J) are quoted in Hertz (Hz). Spectra are referenced internally to residual proton solvent resonances or natural-abundance carbon resonances. IR spectra were recorded on a JASCO FT/IR-4100 spectrometer using an ATR unit. MALDI-TOF mass spectra were recorded on a Bruker Daltronik GmbH (autoflex II) mass spectrometer. High resolution ESI-TOF mass spectrometry was carried out on a microTOF focus instrument (Bruker Daltronik GmbH). For cyclic and square wave voltammetry, a standard commercial electrochemical analyzer (EC epsilon; BAS Instruments, UK) with a three electrode single-compartment cell was used. The supporting electrolyte tetrabutylammonium hexafluorophosphate (TBAHFP) was recrystallized from ethanol/water. The measurements were carried out using

ferrocene/ferrocenium (Fc/Fc^{+}) as an internal standard for the calibration of the potential. Ag/AgCl reference electrode was used. A Pt disc and a Pt wire were used as working and auxiliary electrodes, respectively. The set-up for spectroelectrochemistry consists of a cylindrical quartz cell with an optically transparent and polished bottom, a platinum disc working electrode (6 mm diameter), a gold-coated metal plate as counter electrode and an Ag/AgCl pseudo-reference electrode. An EG & G Princeton Applied Research Model 283 potentiostat was used. UV/vis spectra (JASCO V-670 UV/vis/NIR spectrometer) were recorded in reflection at the polished working electrode, with 100 µm distance between the cell bottom and the surface of the working electrode (adjusted with a micrometer screw). Elemental analyses were performed with an Elementar vario micro cube. DFT calculations for a simplified structure of PBI dianion **4** (fluorinated alkyl chains were replaced by methyl groups and sodium cations are omitted) were performed by using the Gaussian 09 program package[S3] with B3-LYP[S4] as functional and 6-31+G* as basis set. The structures were geometry optimized, followed by frequency calculations on the optimized structures which confirmed the existence of a minimum. For the starting geometry, the coordinates of the single crystal structure analysis were used. The HOMO of the PBI dianion shown in Scheme 1 (main text) was simulated with the help of the GaussView 5 visualization software package[S5] using the data obtained from DFT calculations.

2. Synthesis

Cl_4PBI 1[S6]

Synthesis was carried out according to literature.[S6]

Yield: 309 mg (346 µmol, 77%) orange solid. **^{1}H-NMR** (400 MHz, $CDCl_3$): δ = 5.04 (t, J = 15.2 Hz, 4H, CH_2), 8.75 (s, 4H, CH) ppm. **MS** (MALDI, negative mode, DCTB): m/z calculated for

$C_{32}H_8Cl_4F_{14}N_2O_4$: 891.90 $[M]^-$; found 891.97. **UV/Vis** (CH_2Cl_2, 2×10^{-5} M, 298 K): λ_{max}/nm (ε/$M^{-1}cm^{-1}$) = 520 (36100), 486 (25000), 427 (11300). **Mp.:** >300 °C.

Br_4Cl_4PBI 2

A solution of 1.00 g (1.12 mmol) Cl_4PBI **1** and 1.12 g (3.92 mmol) DBI in 40 mL of Oleum (20% SO_3) was stirred for 18 h at 100 °C. After cooling to room temperature the mixture was poured slowly onto 150 g of ice. 150 mL water were added and the precipitate was collected by filtration, washed several times with water and dried in vacuum. The crude product was purified by column chromatography using dichloromethane as the eluent to give a red solid.

Yield: 667 mg (552 µmol, 51%). **^{1}H NMR** (400 MHz, $CDCl_3$): δ = 5.13 (m, 4H, CH_2) ppm. **^{13}C NMR** (100 MHz, $CDCl_3$): δ = 39.4 ($^2J_{CF}$ = 21.4 Hz, CH$_2$), 121.7 (C_q), 125.5 (C_q), 128.1 (C_q), 129.7 (C_q), 132.2 (C_q), 139.5 (C_q), 160.0 (C_q) ppm. **^{19}F NMR** (376 MHz, $CDCl_3$): –80.4 (t, $^3J_{FF}$ = 9.4 Hz, 6F, CF_3), –116.0 (m, 4F, CF_2), –127.5 (m, 4F, CF_2) ppm. **HRMS** (ESI, positive mode, Acetonitrile/Chloroform 1/1): m/z calculated for $C_{32}H_5Br_4Cl_4F_{14}N_2O_4$: 1208.5438 $[M+H]^+$; found 1208.5427. **Elemental analysis:** calculated (%) for $C_{32}H_4Br_4Cl_4F_{14}N_2O_4$: C 31.82, H 0.33, N 2.32; found C 32.09, H 0.43, N 2.53. **UV/Vis** (CH_2Cl_2, 2×10^{-5} M, 298 K): λ_{max}/nm (ε/$M^{-1}cm^{-1}$) = 523 (29900), 493 (24200), 461 (24700). **Fluorescence** (CH_2Cl_2): λ_{max}/nm = 573 (Φ_{fl} = 2%). **CV** (CH_2Cl_2, 0.1 M TBAHFP, vs. Fc/Fc$^+$): $E_{1/2}$(PBI/PBI$^-$) = –0.49 V, $E_{1/2}$(PBI$^-$/PBI^{2-}) = –0.67 V. **Mp.:** >300 °C.

CN_4Cl_4PBI 3

Synthesis was carried out similar to a literature known procedure.[S7]

300 mg (248 µmol) Br_4Cl_4PBI **2** and 133 mg (1.49 mmol) copper cyanide were mixed in 15 mL of dry DMF under argon and heated to 140 °C for 2.5 h. After cooling to room temperature the mixture was poured onto 150 mL of water. The precipitate was filtered off, washed with water and dissolved in dichloromethane. The solution was dried over $MgSO_4$ and concentrated under reduced pressure. The crude product was purified several times by column chromatography (silica, dichloromethane/ethyl acetate (95/5)) to give an orange-red solid.

Yield: 86.4 mg (87.1 µmol, 35%). **^{1}H NMR** (400 MHz, D_2SO_4): δ = 5.10 (m, 4H, CH_2) ppm. **^{19}F NMR** (376 MHz, D_2SO_4): –80.0 (t, $^3J_{FF}$ = 8.1 Hz, 6F, CF_3), –115.3 (m, 4F, CF_2), –126.9 (m, 4F, CF_2) ppm. **HRMS** (ESI, negative mode, Acetone): m/z calculated for $C_{36}H_4Cl_4F_{14}N_6O_4$: 989.88301 $[M]^-$; found 989.88244. **Elemental analysis:** calculated (%) for $C_{36}H_4Cl_4F_{14}N_6O_4$: C

43.58, H 0.41, N 8.47, found C 44.01, H 0.44, N 8.27. **UV/Vis** (CH_2Cl_2, 2×10^{-5} M, 298 K): λ_{max}/nm (ε/$M^{-1}cm^{-1}$) = 528 (34100), 494 (22000), 447 (14900). **Fluorescence** (CH_2Cl_2): λ_{max}/nm = 559 (Φ_{fl} = 11%). **CV** (CH_2Cl_2, 0.1 M TBAHFP, vs Fc/Fc^+): $E_{1/2}$(PBI/PBI^{-}) = –0.07 V, $E_{1/2}$(PBI^{-}/PBI^{2-}) = –0.41 V. **Mp.:** >300 °C decomposition.
Due to the low solubility of this compound in all organic solvents as well as in D_2SO_4 no ^{13}C resonances could be detected.

CN_4Cl_4PBI dianion disodium salt 4

20 mg (20.2 µmol) of CN_4Cl_4PBI **3**, 20 mg (238 µmol) $NaHCO_3$ and 2 mg (10%) Pd/C were mixed in 10 mL of dry methanol under nitrogen atmosphere. After replacing nitrogen by hydrogen, a colour change from orange to blue can be observed within few minutes. After stirring the solution for additional 4 h at room temperature the catalyst was filtered off through a pad of celite. After removing the solvent under reduced pressure, the residue was dissolved in 20 mL acetone and the product was precipitated by addition of 100 mL pentane. This mixture was cooled to 5 °C and the product was filtered off, washed with pentane and dried under vacuum at 50 °C.
Yield: 17.3 mg (16.1 µmol, 80%) blue solid. **^{1}H NMR** (400 MHz, Acetone-D_6): δ = 5.07 (broad s, 4H, CH_2) ppm. **^{13}C NMR** (100 MHz, Acetone-D_6): δ = 38.9 (t, $^2J_{CF}$ = 21.2 Hz, CH_2), quaternary carbon atoms and CF_2, CF_3 groups could not be detected. **^{19}F NMR** (376 MHz, Acetone-D_6): –81.4 (t, $^3J_{FF}$ = 9.7 Hz, 6F, CF_3), –115.8 (m, 4F, CF_2), –128.5 (m, 4F, CF_2) ppm. **HRMS** (ESI, negative mode, Acetone): *m*/*z* calculated for $C_{36}H_4Cl_4F_{14}N_6O_4$: 989.88301 $[M]^-$; found 989.88361. Sodium cations cannot be detected by this method. **Elemental analysis:** calculated (%) for $Na_2[C_{36}H_4Cl_4F_{14}N_6O_4]\cdot 2H_2O$: C 40.25, H 0.75, N 7.82; found: C 40.67, H 0.94, N 7.58. **UV/Vis** (Acetone, 2×10^{-5} M, 298 K): λ_{max}/nm (ε/$M^{-1}cm^{-1}$) = 793 (91300), 718 (22400). **CV** (Acetone, 0.1 M TBAHFP, vs Fc/Fc^+): $E_{1/2}$(PBI/PBI^{-}) = –0.15 V, $E_{1/2}$(PBI^{-}/PBI^{2-}) = –0.35 V.

3. Spectroelectrochemical measurements and UV-Vis-NIR absorption spectra

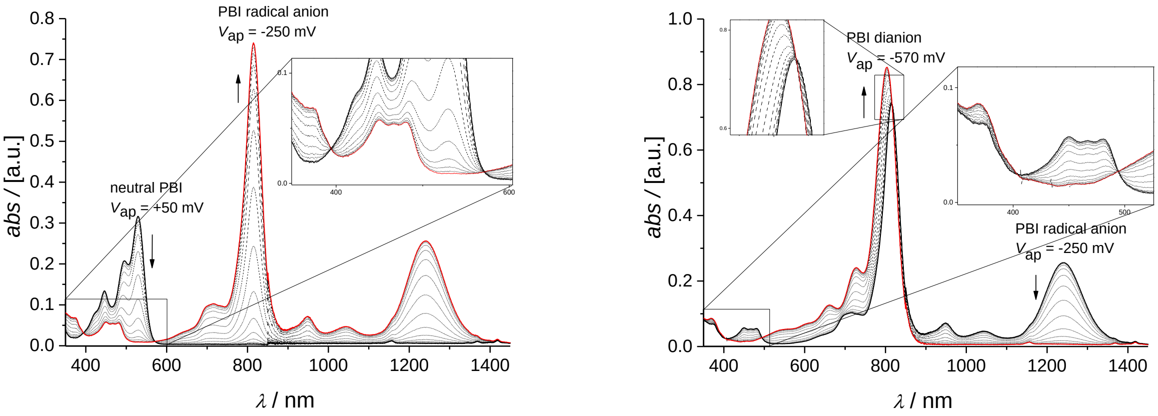

Fig. S1 Left: UV-vis-NIR absorption changes upon electrochemical reduction of PBI **3** to PBI $\mathbf{3}^{\bullet-}$ (in steps of 20 mV) at potentials from +50 to –250 mV. Right: UV-Vis-NIR absorption changes upon electrochemical reduction of PBI $\mathbf{3}^{\bullet-}$ to PBI $\mathbf{3}^{2-}$ (in steps of 20 mV) at potentials from –250 to –570 mV. Inset: magnification of the isosbestic points at 394 and 572 nm (PBI **3** to PBI $\mathbf{3}^{\bullet-}$) and at 404, 494 and 816 nm (PBI $\mathbf{3}^{\bullet-}$ to PBI $\mathbf{3}^{2-}$). 0.2 M TBAHFP, DCM, 25 °C.

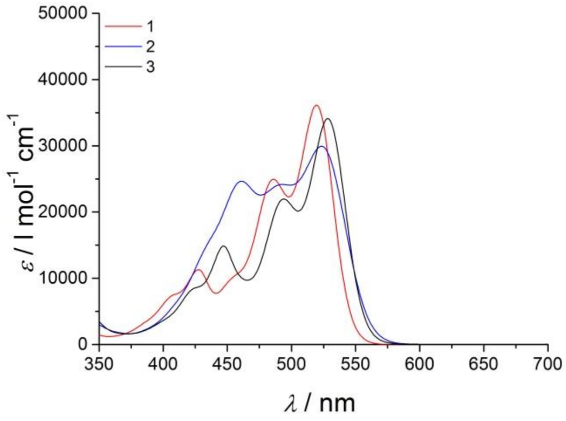

Fig. S2 UV-vis absorption spectra of PBIs **1**, **2**, **3** in dichloromethane, 25 °C, $c = 2\times10^{-5}$ M.

4. CV and SW data

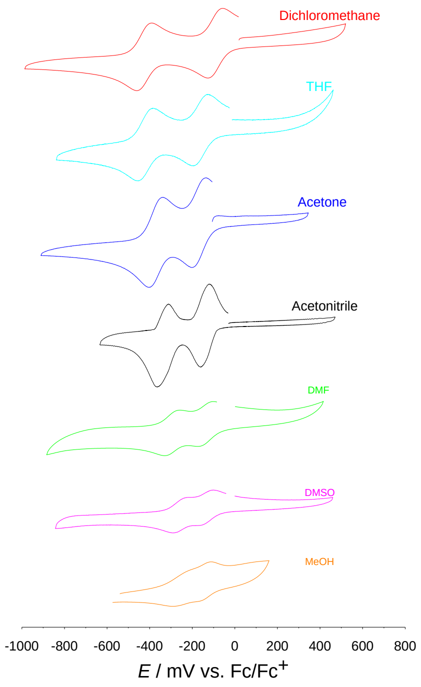

Fig. S3 Cyclic voltammograms of PBI **3** in solvents of different polarity. The dianion disodium salt **4** has been used in the case of MeOH for solubility reasons. Reference electrode: Ag/AgCl, working and auxiliary electrode: Pt. 0.1 M TBAHFP, Fc/Fc^+, 25 °C, $c \sim 2\times10^{-4}$ M.

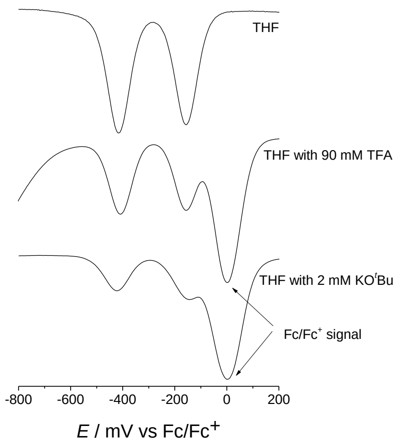

Fig. S4 Square wave voltammograms of PBI **3** in THF, THF acidified with trifluoroacetic acid, THF basic with KO^{t}Bu. Reference electrode: Ag/AgCl, working and auxiliary electrode: Pt. 0.1 M TBAHFP, Fc/Fc$^+$, 25 °C, c~2×10^{-4} M.

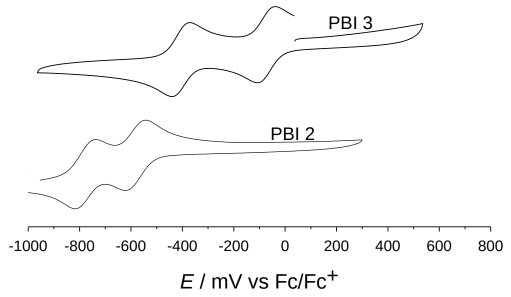

Fig. S5 Cyclic voltammograms of PBI **3** and PBI **2** in DCM, Reference electrode: Ag/AgCl, working and auxiliary electrode: Pt. 0.1 M TBAHFP, Fc/Fc$^+$, 25 °C, c~2×10^{-4} M.

Table S1. Reduction potentials and LUMO levels PBIs containing heptafluorobutyl chains at the imide positions.

**PBI**	$E_{1/2}$ **(PBI/PBI$^{\bullet-}$)/V**	$E_{1/2}$ **(PBI$^{\bullet-}$/PBI^{2-})/V**	[b]E_{LUMO}**/eV**
[a]***N*,*N*'-Di(heptafluorobutyl)-3,4:9,10-tetracarboxylic acid bisimide**	–0.95	–1.15	–3.85
[a]**1**	–0.74	–0.95	–4.06
**2**	–0.49	–0.67	–4.31
**3**	–0.07	–0.41	–4.73

Half wave potentials were determined by cyclic voltammetry measured in 0.1 M TBAHFP in DCM vs. Fc/Fc^+ a) Data from literature[S6]; b) energy of the lowest unoccupied molecular orbital E_{LUMO} in eV against vacuum was calculated according to literature methods from $E_{1/2}$ (PBI/PBI$^{\bullet-}$) by $E_{\mathrm{LUMO}} = -[E_{1/2}(\mathrm{PBI/PBI}^{\bullet-}) + 4.8\ \mathrm{eV}]$ and the value for ferrocene below vacuum (–4.8 eV).[S8]

5. **Single crystal data**

Single crystals suitable for X-ray diffraction experiments were grown by slow diffusion of pentane into a 1mM solution of **4** in acetone at room temperature.

Crystal Data were collected on a Bruker D8Quest Kappa Diffractometer using CuKα-radiation from an Incoatec IμS microsource with Montel multi layered mirror, a Photon100 CMOS detector and Apex2 software at 100K. The structure was solved using direct methods (SHELXS), expanded with Fourier techniques and refined with SHELXL. All non-hydrogen atoms were refined anisotropically. Hydrogen atoms were included in the structure factor calculation on geometrically idealized positions.

Crystal data: $C_{89.40}H_{44.52}Cl_8F_{28}N_{12}Na_4O_{14.43}$, M = 2425.21, blue needle: 0.307 x 0.156 x 0.095 mm^3, triclinic P-1, *a*: 14.6741(9) Å, *b*: 15.8676(10) Å, *c*: 24.1918(15) Å, *α*: 91.946(2) °, *β*: 106.327(2) °, *γ*: 115.228(2) °, cell volume: 4812.8(5) Å^3, *Z*: 2, λ = 1.54178 Å, *T*=100K, ρcalc = 1.674 g/cm^3, F_{000}: 2425, μ: 3.447 mm^{-1}, R_1: 0.0485, R_{wt}: 0.1285, GooF: S = 1.016, 19566 refls (R_{int}) = 0.0338, 19566 refls total, 1669 params, 527 restraints.

Crystallographic data have been deposited with the Cambrige Crystallographic Data Centre as supplementary publication no. CCDC 1032959. These data can be obtained free of charge from The Cambrige Crystallographic Data Centre *via* www.ccdc.ac.uk/data.request/cif.

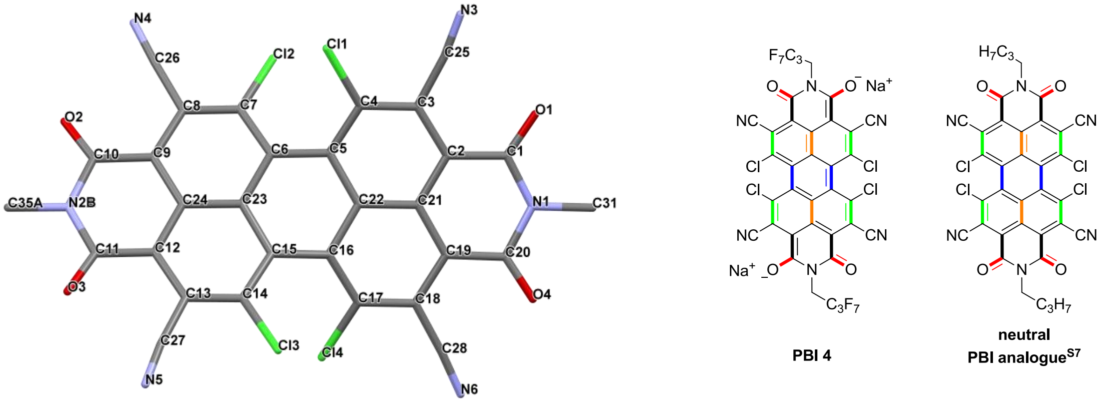

Fig. S6 Left: Molecular structure of $[CN_4Cl_4PBI]^{2-}$ in the solid state (fluorinated alkyl chains, counter ions and solvent molecules have been omitted for clarity). Schematic illustration of the most deviating bond length of PBI **4** compared to a neutral tetracyanotetrachloro PBI analogue containing *n*-butyl chains at the imide positions that was synthesized in the group of Wang.[S7]

Table S2. Comparison of bond distances and torsion angles of the PBI dianion disodium salt **4** with bond distances and angles of a neutral PBI analogue containing *n*-butyl chains at the imide positions (Fig. S6).[S7]

	**PBI dianion disodium salt 4**	***n*-butyl-substituted CN_4Cl_4PBI analogue (Fig. S6)**[S7]
**C=O**	1.224–1.240 Å	1.189–1.229 Å
**C=C** (C15-C16, C5-C6)	1.429–1.437 Å	1.478–1.484 Å
**C=C** (C3-C4,C17-C18, C7-C8, C13-C14)	1.378–1.382 Å	1.401–1.449 Å
**C=C** (C21-C22, C23-C24)	1.433–1.439 Å	1.392–1.425 Å
**C-C** (C1-C2, C9-C10, C11-C12, C19-C20)	1.437–1.446 Å	1.470–1.500 Å
C-C (C2-C3, C8-C9, C12-C13, C18-C19)	1.417–1.431 Å	1.380–1.392 Å
C-C (C9-C24, C12-C24, C2-C21, C19-C21)	1.409–1.412 Å	1.377–1.438 Å
C-C (C4-C5, C6-C7, C14-C15, C16-C17)	1.421–1.431 Å	1.361–1.400 Å
C-C (C6-C23, C15-C23, C5-C22, C16-C22)	1.423–1.430 Å	1.396–1.440 Å
C-N (in imide rings)	1.395–1.403 Å	1.385–1.416 Å
C-Cl	1.726–1.735 Å	1.716–1.729 Å
C-C (to CN groups)	1.436–1.446 Å	1.421–1.470 Å
CN triple bond	1.144–1.151 Å	1.119–1.174 Å
torsion angle	32.9° to 33.9°	35.0° to 36.4°

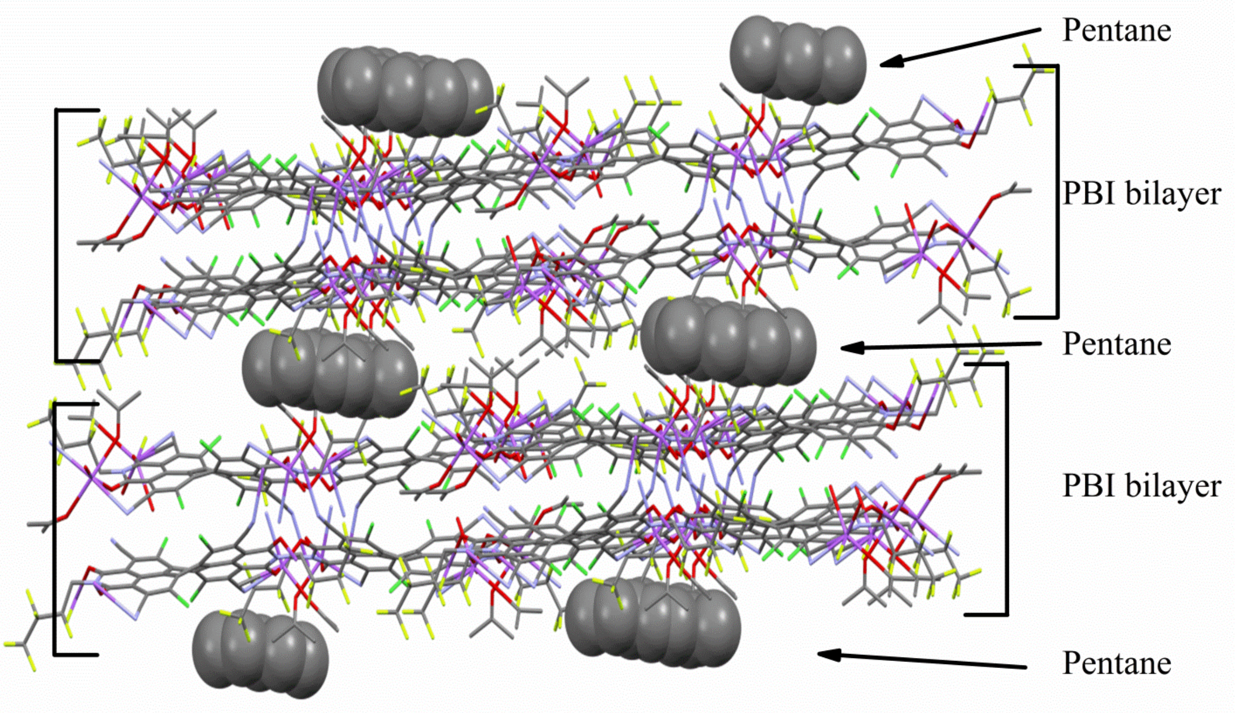

Fig. S7 Molecular packing of PBI **4** within the solid state. Pentane molecules are incorporated between ever PBI bilayer.

6. **IR spectra**

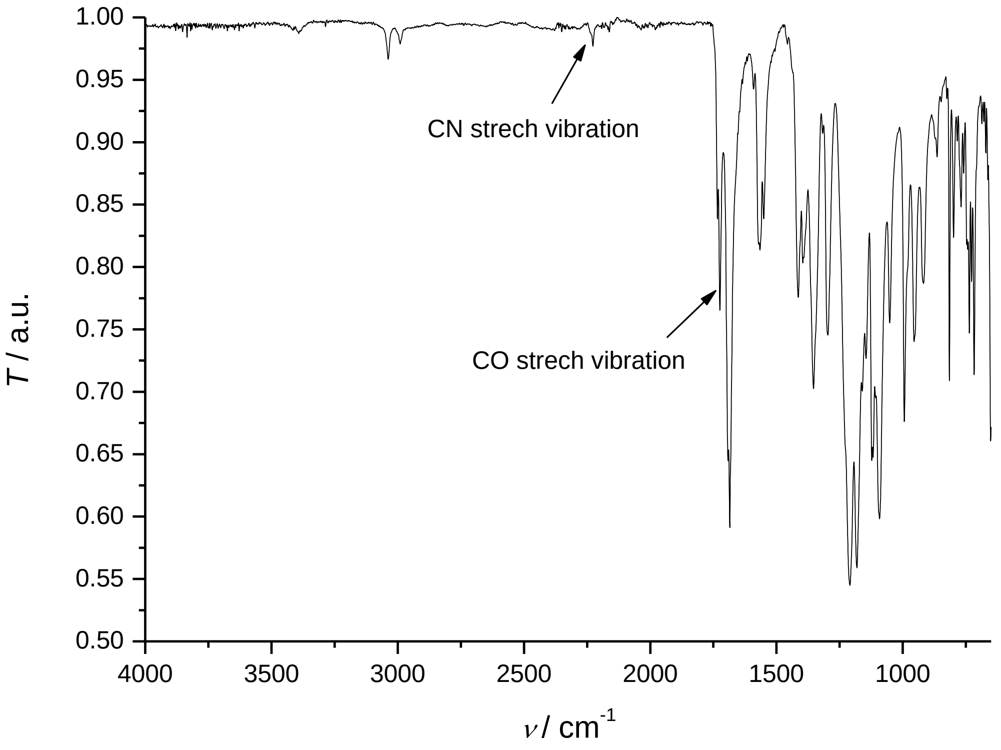

Fig. S8 IR spectrum (transmission) of compound **3** in the range of 650 up to 4000 cm^{-1}.

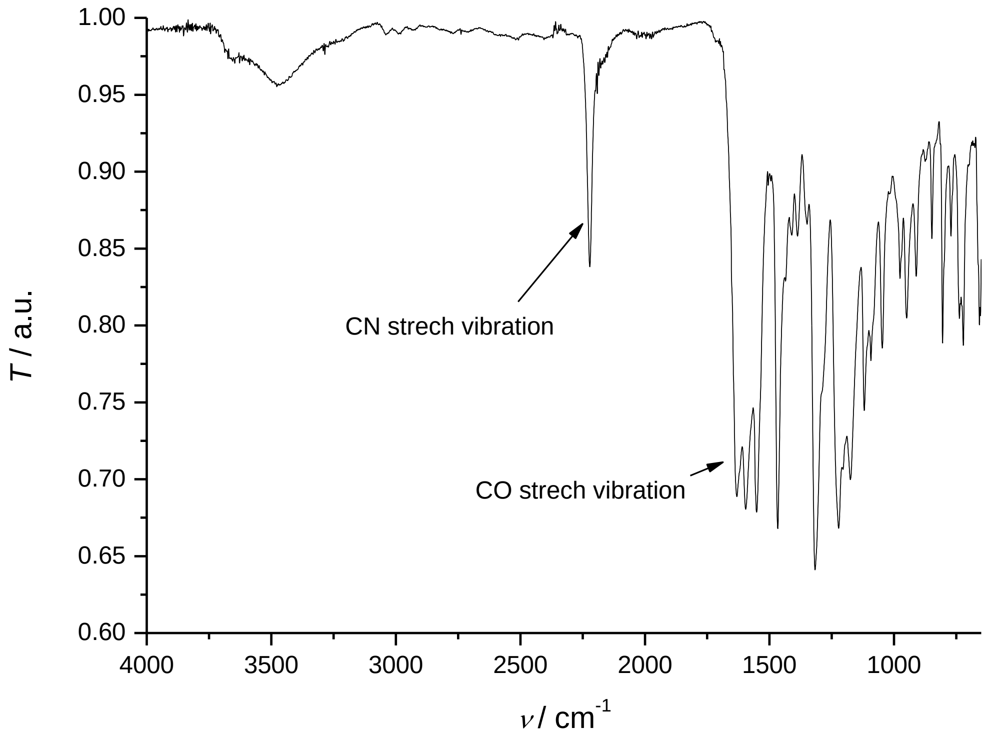

Fig. S9 IR spectrum (transmission) of the PBI dianion disodium salt **4** in the range of 650 up to 4000 cm^{-1}.

7. ^{1}H and ^{13}C NMR

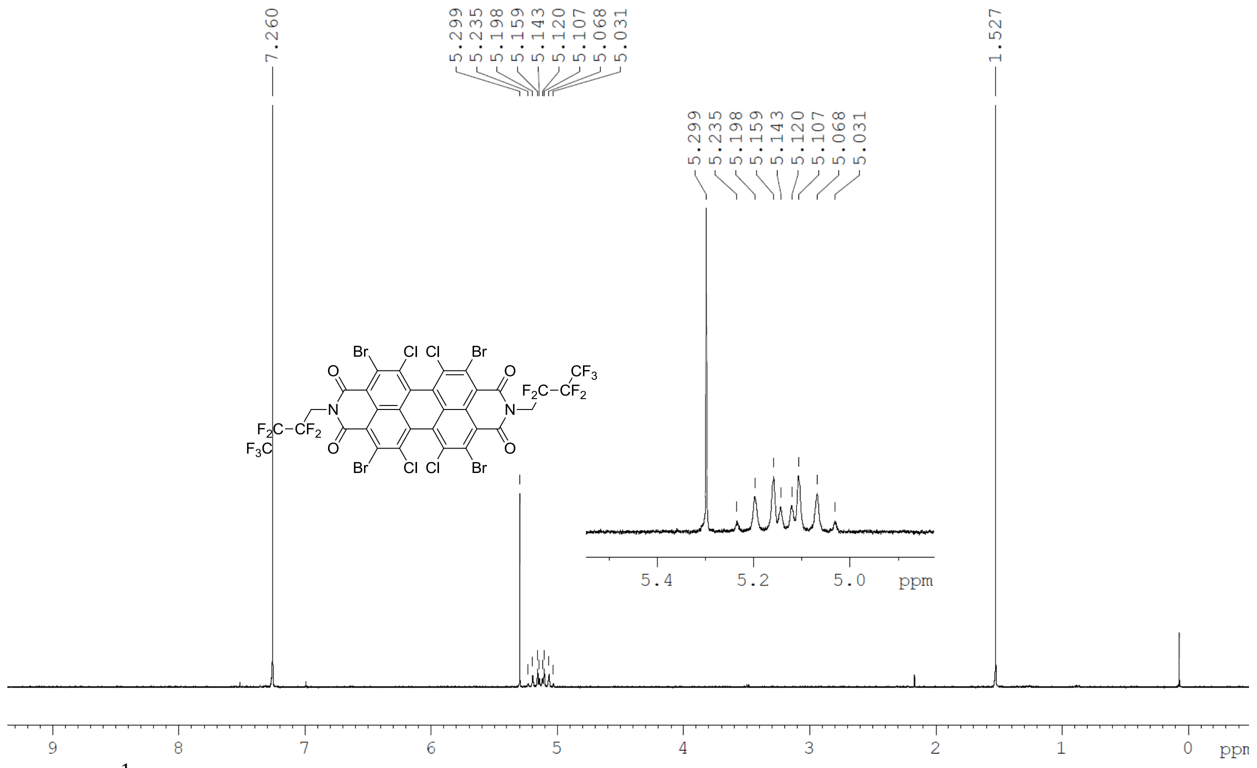

Fig. S10 ^{1}H NMR spectrum of Br_4Cl_4PBI **2** in $CDCl_3$ (7.26 ppm) with residual CH_2Cl_2 at 5.30 ppm and H_2O at 1.53 ppm.

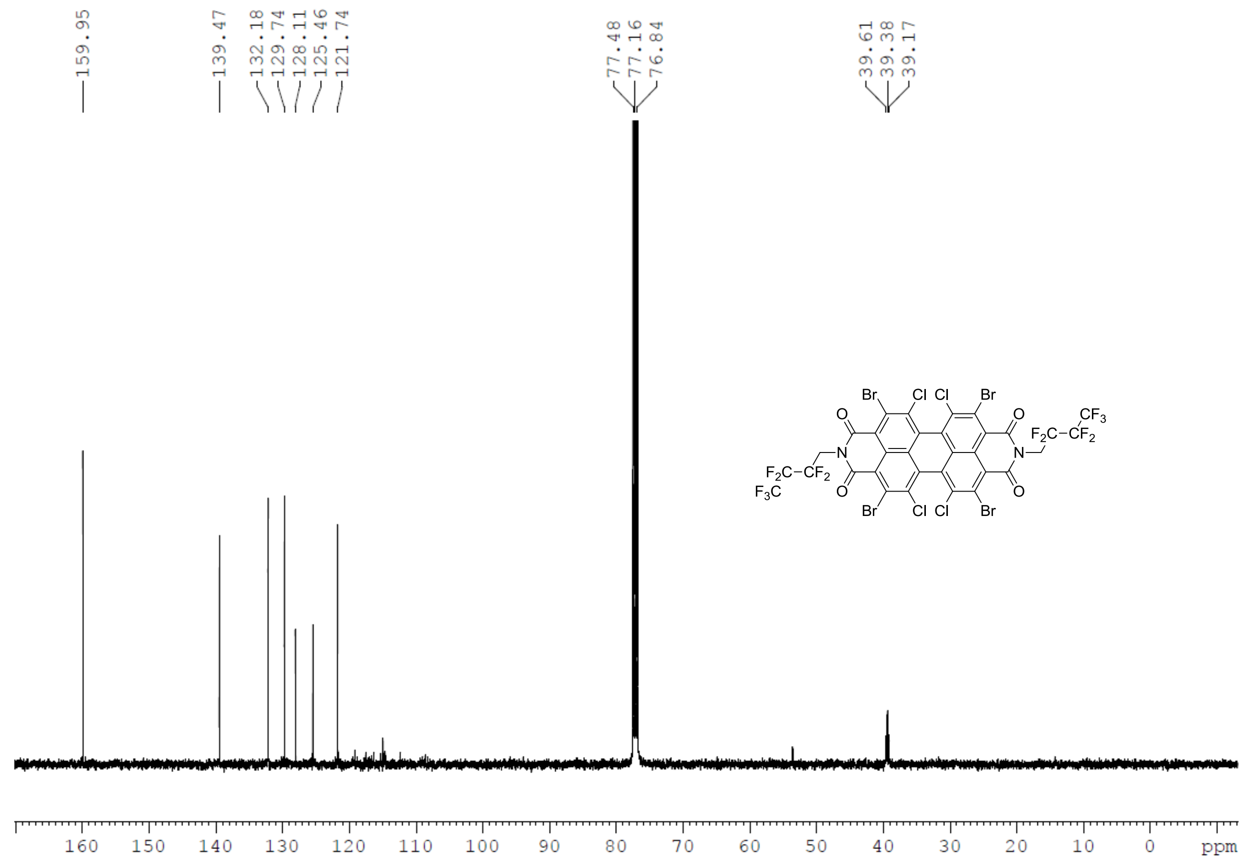

Fig. S11 ^{13}C NMR spectrum of Br_4Cl_4PBI **2** in $CDCl_3$ (77.16 ppm); only the quaternary resonances could be detected. The N$C$$H_2$ carbon atoms give rise to a triplet at 39.38 ppm within the aliphatic region; CF_2 and CF_3 could not be detected.

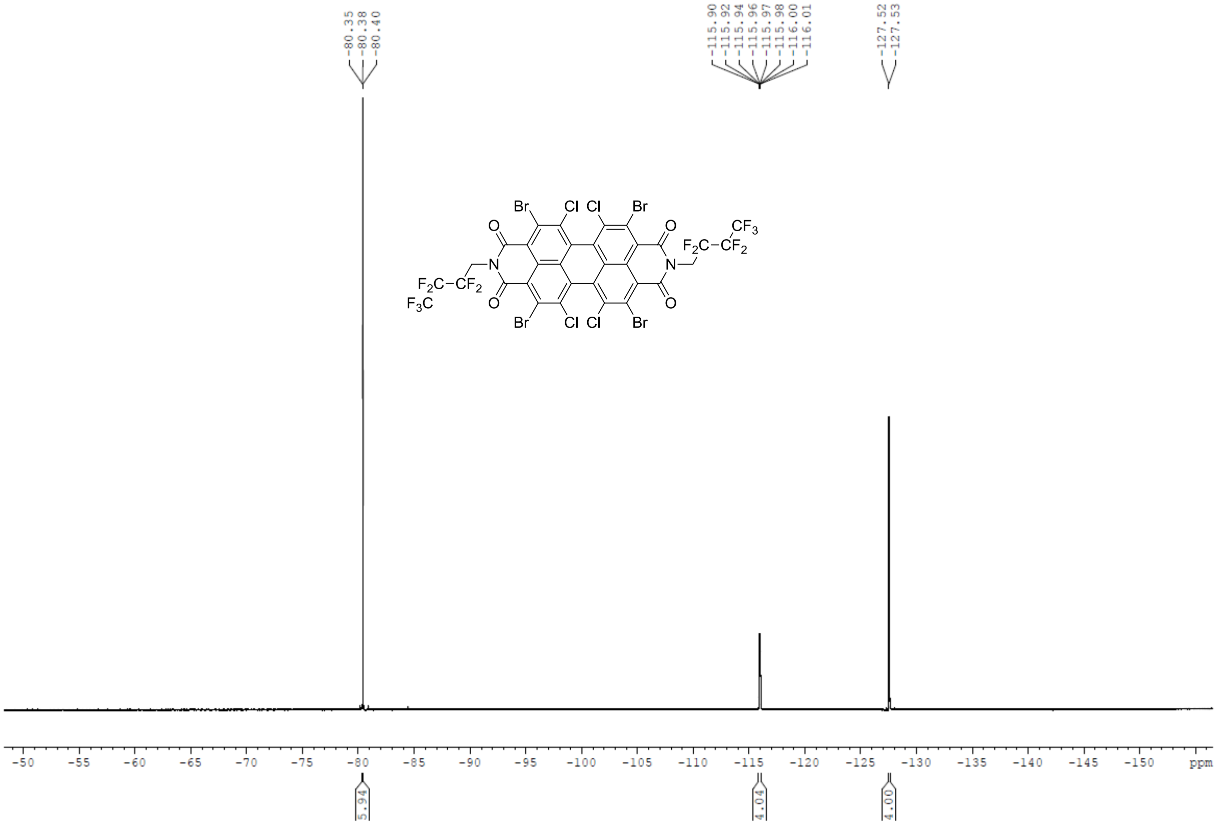

Fig. S12 ^{19}F NMR spectrum of Br_4Cl_4PBI **2** in $CDCl_3$.

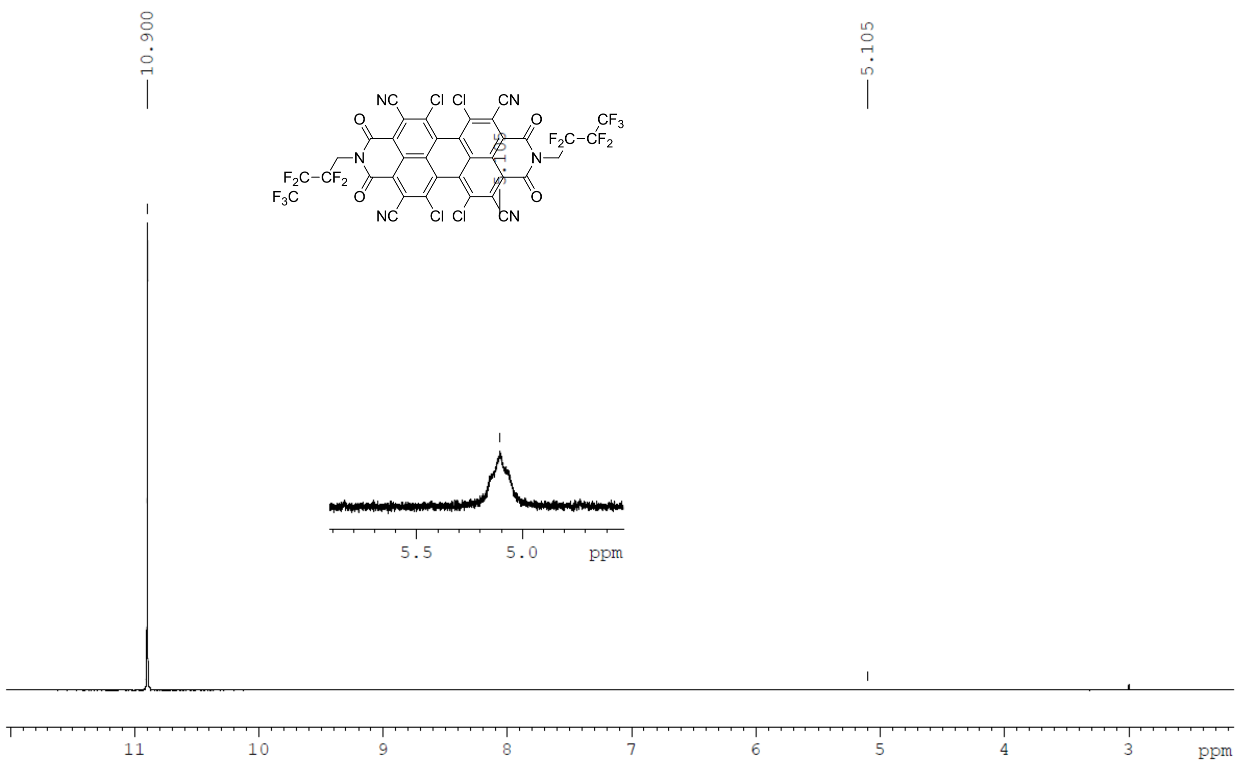

Fig. S13 ^{1}H NMR spectrum of $(CN)_4Cl_4$PBI **3** in D_2SO_4 (10.90 ppm) with residual H_2O at 3.00 ppm.

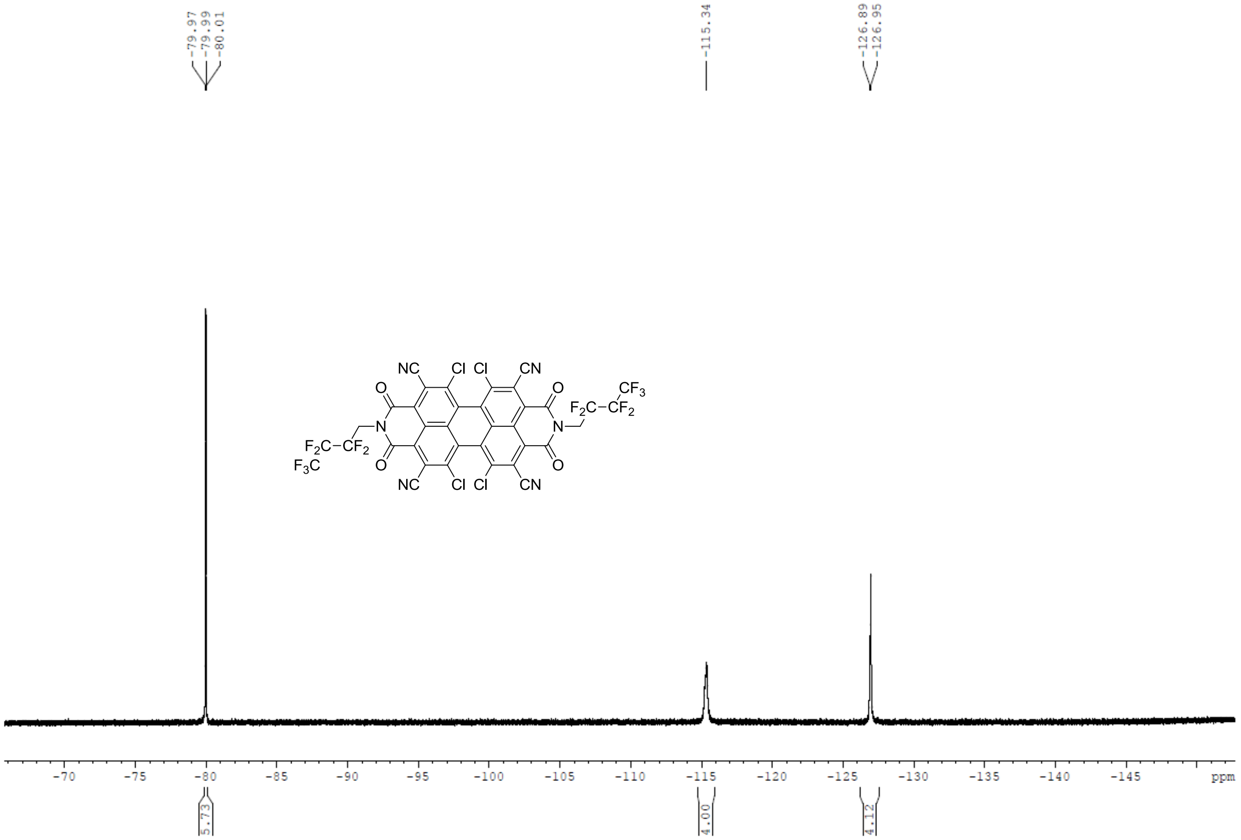

Fig. S14 ^{19}F NMR spectrum of $(CN)_4Cl_4PBI$ **3** in D_2SO_4.

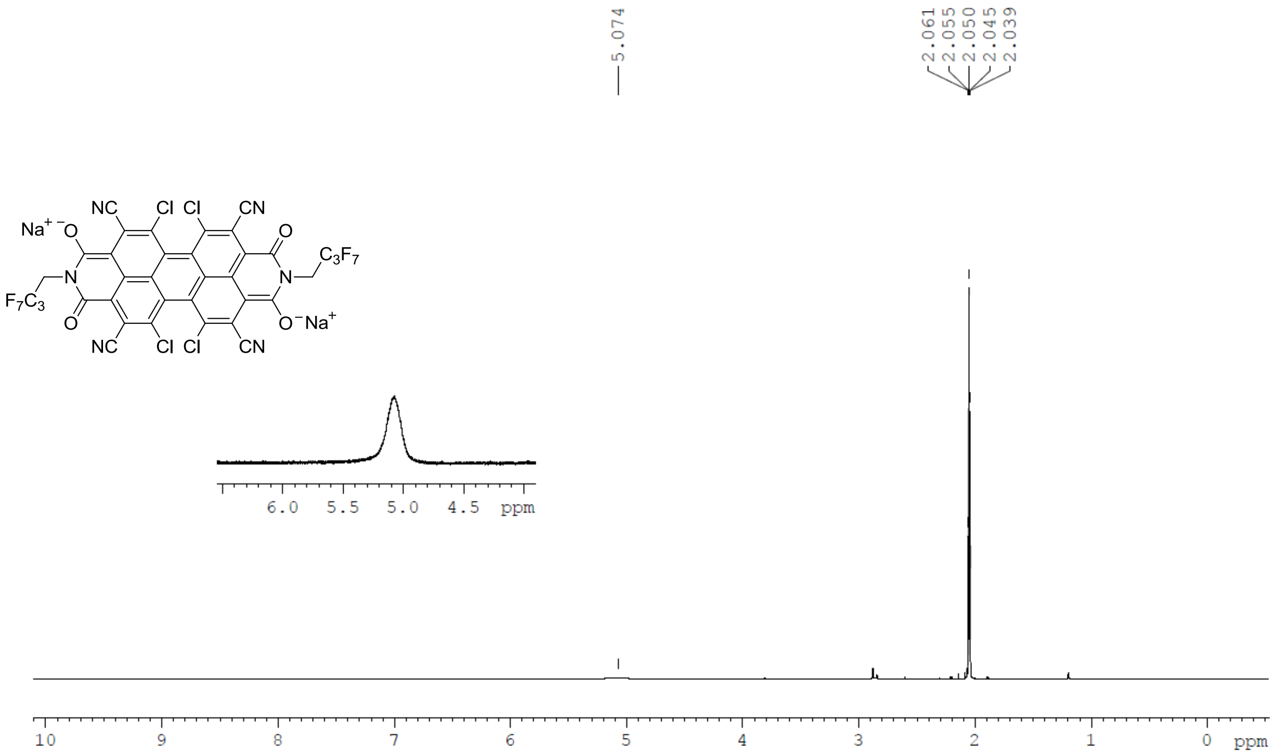

Fig. S15 ^{1}H NMR spectrum of PBI dianion disodium salt **4** in d_6-acetone (2.05 ppm) with residual H_2O at 2.88 ppm.

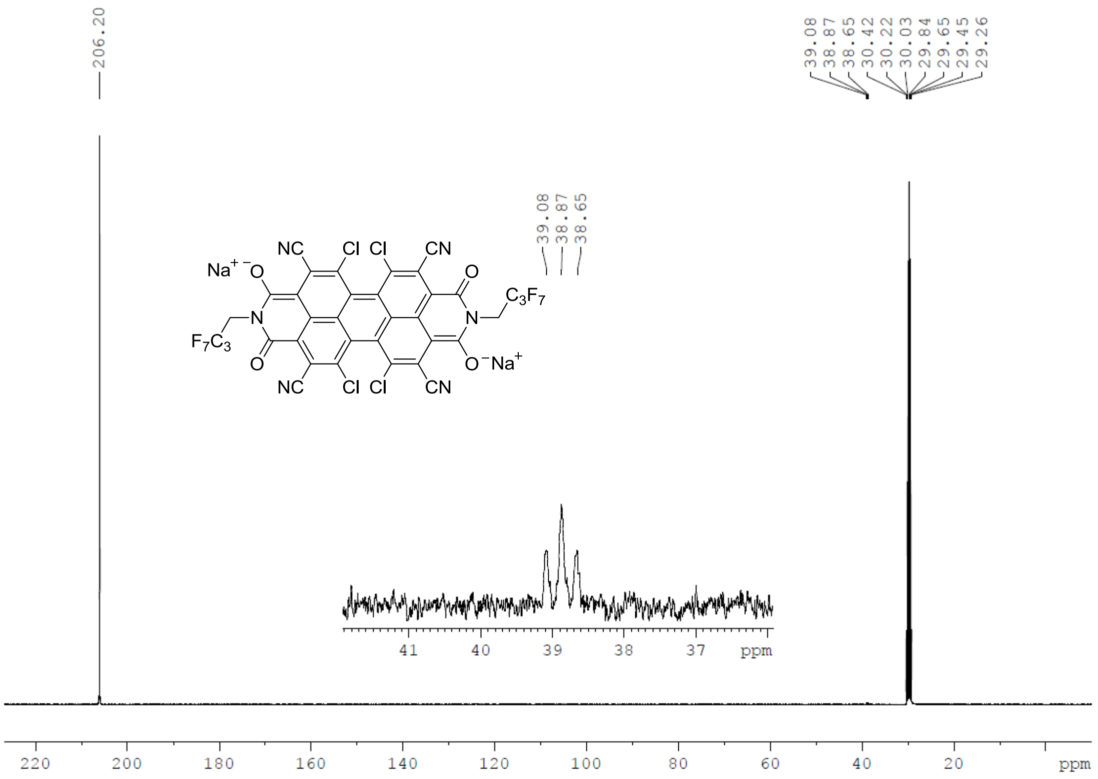

Fig. S16 ^{13}C NMR spectrum of PBI dianion disodium salt **4** in d_6-acetone (29.84 and 206.20 ppm). The N*C*H_2 carbon atoms give rise to a triplet at 38.87 ppm within the aliphatic region. Quaternary carbon atoms and *C*F_2, *C*F_3 groups could not be detected.

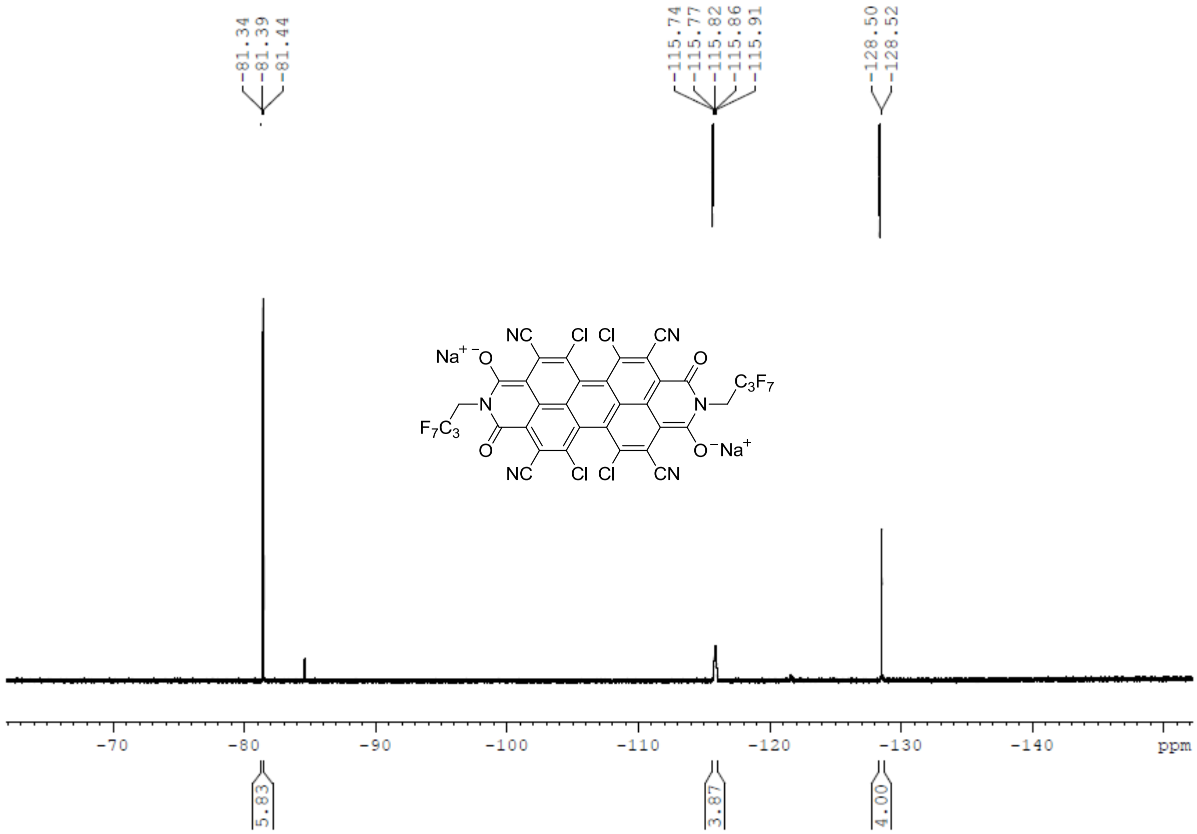

Fig. S17 ^{19}F NMR spectrum of the PBI dianion disodium salt **4** in d_6-acetone.

9. **HRMS spectra**

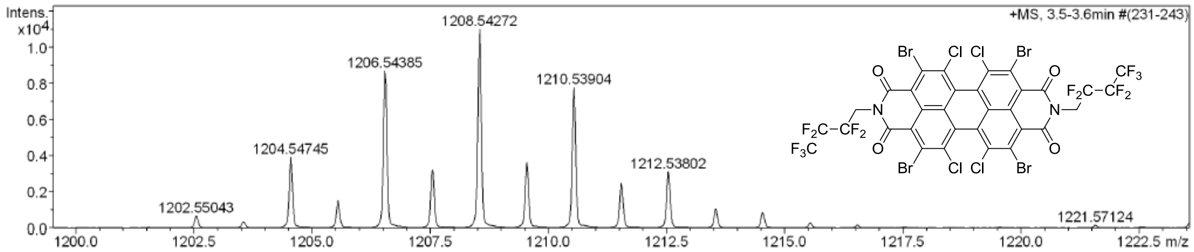

Fig. S18 ESI-MS spectrum of Br_4Cl_4PBI **2** (positive mode, acetonitrile/chloroform 1/1) $[M+H]^+$.

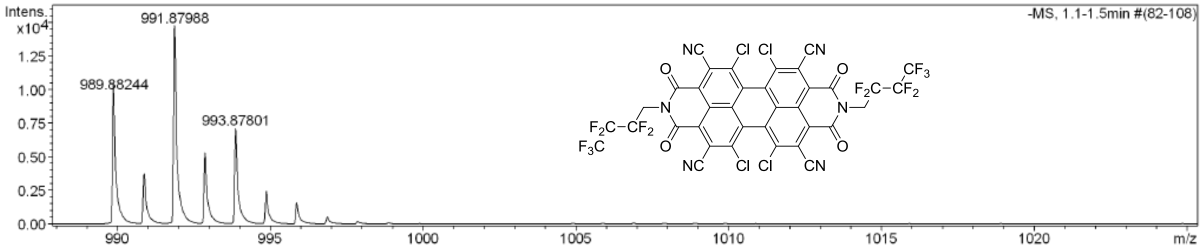

Fig. S19 ESI-MS spectrum of CN_4Cl_4PBI **3** (negative mode, acetone) $[M]^-$.

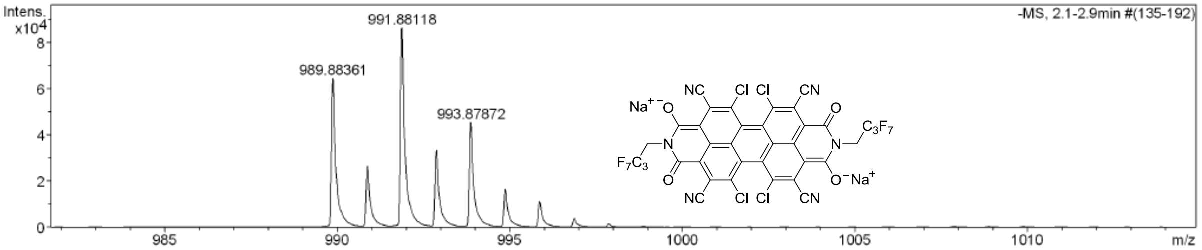

Fig. S20 ESI-MSspectrum of PBI dianion disodium salt **4** (negative mode, acetone) $[M\text{-}2Na]^-$.

10. **References**

S1 W. Gottardi, *Monatsh. Chem.,* 1967, **98**, 507.

S2 J. R. Lakowicz, *Principles of Fluorescence Spectroscopy,* 2nd ed., Kluwer Academic/Plenum, New York, 1999.

S3 Gaussian 09, Revision A.02, M. J. Frisch, G. W. Trucks, H. B. Schlegel, G. E. Scuseria, M. A. Robb, J. R. Cheeseman, G. Scalmani, V. Barone, B. Mennucci, G. A. Petersson, H. Nakatsuji, M. Caricato, X. Li, H. P. Hratchian, A. F. Izmaylov, J. Bloino, G. Zheng, J. L. Sonnenberg, M. Hada, M. Ehara, K. Toyota, R. Fukuda, J. Hasegawa, M. Ishida, T. Nakajima, Y. Honda, O. Kitao, H. Nakai, T. Vreven, J. A. Montgomery, Jr., J. E. Peralta, F. Ogliaro, M. Bearpark, J. J. Heyd, E. Brothers, K. N. Kudin, V. N. Staroverov, R. Kobayashi, J. Normand, K. Raghavachari, A. Rendell, J. C. Burant, S. S. Iyengar, J. Tomasi, M. Cossi, N. Rega, J. M. Millam, M. Klene, J. E. Knox, J. B. Cross, V. Bakken, C. Adamo, J. Jaramillo, R. Gomperts, R. E. Stratmann, O. Yazyev, A. J. Austin, R. Cammi, C. Pomelli, J. W. Ochterski, R. L. Martin, K. Morokuma, V. G. Zakrzewski, G. A. Voth, P. Salvador, J. J. Dannenberg, S. Dapprich, A. D. Daniels, Ö. Farkas, J. B. Foresman, J. V. Ortiz, J. Cioslowski, and D. J. Fox, Gaussian, Inc., Wallingford CT, 2009.

S4 (a) A. D. Becke, *Phys. Rev. A, 1988*, **38**, 3098; (b) C. Lee, W. Yang and R. G. Parr, *Phys. Rev. B, 1988*, **37**, 785; (c) A. D. Becke, *J. Chem. Phys., 1993*, **98**, 5648.

S5 GaussView, Version 5, R. Dennington, T. Keith, J. Millam, *Semichem. Inc*., Shawnee Mission KS, 2009.

S6 R. Schmidt, J. H. Oh, Y. S. Sun, M. Deppisch, A. M. Krause, K. Radacki, H. Braunschweig, M. Könemann, P. Erk, Z. A. Bao and F. Würthner, *J. Am. Chem. Soc.*, 2009, **131**, 6215.

S7 J. Gao, C. Xiao, W. Jiang and Z. Wang, *Org. Lett.*, 2014, **16**, 394.

S8 (a) I. Seguy, P. Jolinat, P. Destruel, R. Mamy, H. Allouchi, C. Courseille, M. Cotrait and H. Bock, *Chem. Phys. Chem.*, *2001*, **2**, 448; (b) C. M. Cardona, W. Li, A. E. Kaifer, D. Stockdale and G. C. Bazan, *Adv. Mater.*, *2011*, **23**, 2367.
